# Glycemic control modifies LDL-C–DKD risk: a U-shaped association in well-controlled type 2 diabetes

**DOI:** 10.3389/fnut.2025.1660820

**Published:** 2025-09-17

**Authors:** Kaili Zheng, Chaoyong He, Guangming Chen, Huabin Wang, Yongjun Ma

**Affiliations:** ^1^Department of Clinical Laboratory, Affiliated Jinhua Hospital, Zhejiang University School of Medicine, Jinhua, China; ^2^Department of Cardiology, Taihe Hospital, Affiliated Hospital of Hubei University of Medicine, Shiyan, China; ^3^Department of General Practice, Affiliated Jinhua Hospital, Zhejiang University School of Medicine, Jinhua, China

**Keywords:** low-density lipoprotein cholesterol, diabetes kidney disease, type 2 diabetes, U-shaped association, glycemic control

## Abstract

**Background:**

The relationship between low-density lipoprotein cholesterol (LDL-C) levels and diabetic kidney disease (DKD) risk remains controversial, with limited evidence on its interaction with modifiable risk factors. This study aimed to investigate the dose–response relationship between LDL-C and DKD risk in patients with type 2 diabetes (T2D).

**Methods:**

A retrospective cohort of 3,040 patients with T2D without baseline DKD was followed. Association between LDL-C and DKD risk was analyzed using Cox regression analysis, interaction analysis, and restricted cubic splines (RCS). Sensitivity analyses excluded lipid-lowering medication users, and threshold effects were validated using piecewise regression and survival analysis.

**Results:**

A total of 665 (21.9%) patients developed DKD during the follow-up (median: 3.13 years). In the fully adjusted model, LDL-C as a continuous variable showed no significant association with DKD risk (*p* = 0.061). When analyzed by quartiles, the hazard ratios (HRs) displayed a non-monotonic pattern: Compared to Q1, Q2 had the lowest risk (HR = 0.69, *p* = 0.001), followed by a partial rebound in Q3 (HR = 0.80, *p* = 0.046), and a subsequent decline in Q4 (HR = 0.72, *p* = 0.005), suggesting potential non-linearity. A significant LDL-C-by-glycemia control interaction was observed (P_interaction_ = 0.013). In the HbA1c ≤ 7% subgroup, RCS analysis demonstrated a U-shaped relationship between LDL-C and DKD risk (P_non-linear_ < 0.001), with nadir risk observed at 2.66–3.57 mmol/L. The risk increased below 2.66 mmol/L (HR = 1.55, *p* = 0.015) and trended upward above 3.57 mmol/L (HR = 1.47, *p* = 0.121). In this subgroup, sensitivity analyses excluding lipid-lowering drug users confirmed robustness, and survival curves showed lower DKD incidence in the intermediate LDL-C group (2.66–3.57 mmol/L) vs. low/high groups (*p* = 0.004). No associations were found in the HbA1c > 7% subgroup.

**Conclusion:**

Glycemic control modulates the LDL-C–DKD risk association in patients with T2D, with a U-shaped relationship observed in those with good glycemic control, thereby emphasizing the necessity of integrating glycemic status into LDL-C target evaluations.

## 1 Introduction

Diabetic kidney disease (DKD) is one of the most severe microvascular complications of diabetes, with approximately 20–40% of patients with type 2 diabetes (T2D) progressing to DKD (1–3). DKD not only significantly increases the risk of end-stage renal disease but is also closely associated with cardiovascular event-related mortality, posing a major global public health challenge (4, 5). Although strict glycemic and blood pressure control can delay renal function deterioration, some patients still experience DKD progression even after achieving treatment targets (6), suggesting that the potential role of other metabolic factors remains incompletely elucidated.

Abnormal lipid metabolism has recently garnered widespread attention in DKD pathogenesis. Studies indicate that lipids promote DKD progression through mechanisms such as oxidative stress, inflammatory responses, autophagy dysregulation, endoplasmic reticulum stress, and apoptosis, leading to podocyte injury, extracellular matrix deposition, and macrophage infiltration (7–9). Low-density lipoprotein cholesterol (LDL-C), recognized as a core risk factor for atherosclerosis, has long been managed under the traditional paradigm of “the lower, the better” (10, 11). However, the 2024 China HEART project revealed a U-shaped association between LDL-C and all-cause mortality in primary prevention and low-risk populations, suggesting that both elevated and extremely low LDL-C levels may increase mortality risk (12). These findings challenge conventional lipid management strategies. Notably, current clinical studies on LDL-C and DKD risk remain limited and yield conflicting results. Some cohort studies demonstrated a positive correlation between LDL-C levels and albuminuria or estimated glomerular filtration rate (eGFR) decline (13, 14); a multicenter study reported that statins significantly reduced DKD risk by lowering LDL-C, particularly in populations with stringent lipid control (15). Conversely, other clinical studies indicated no statistically significant association between statin-induced LDL-C reduction and DKD cumulative incidence (hazard ratio 0.97, 95% CI 0.85–1.10; *p* = 0.60) (16). Additionally, a Mendelian randomization study found no causal relationship between LDL-C levels and DKD or urinary albumin excretion (17).

These controversies may arise from multiple factors. First, the dose–response relationship between LDL-C and DKD risk may exhibit non-linear characteristics, whereas prior studies predominantly used linear models, potentially failing to fully elucidate intrinsic associations. Second, the relationship may vary due to heterogeneity in metabolic status, such as differing glycemic control levels. Therefore, our study aimed to systematically explore the complex association patterns between LDL-C and DKD risk through a large-scale retrospective cohort analysis, thereby providing a reference for individualized lipid management in T2D patients.

## 2 Materials and methods

### 2.1 Study population

**Figure 1:**
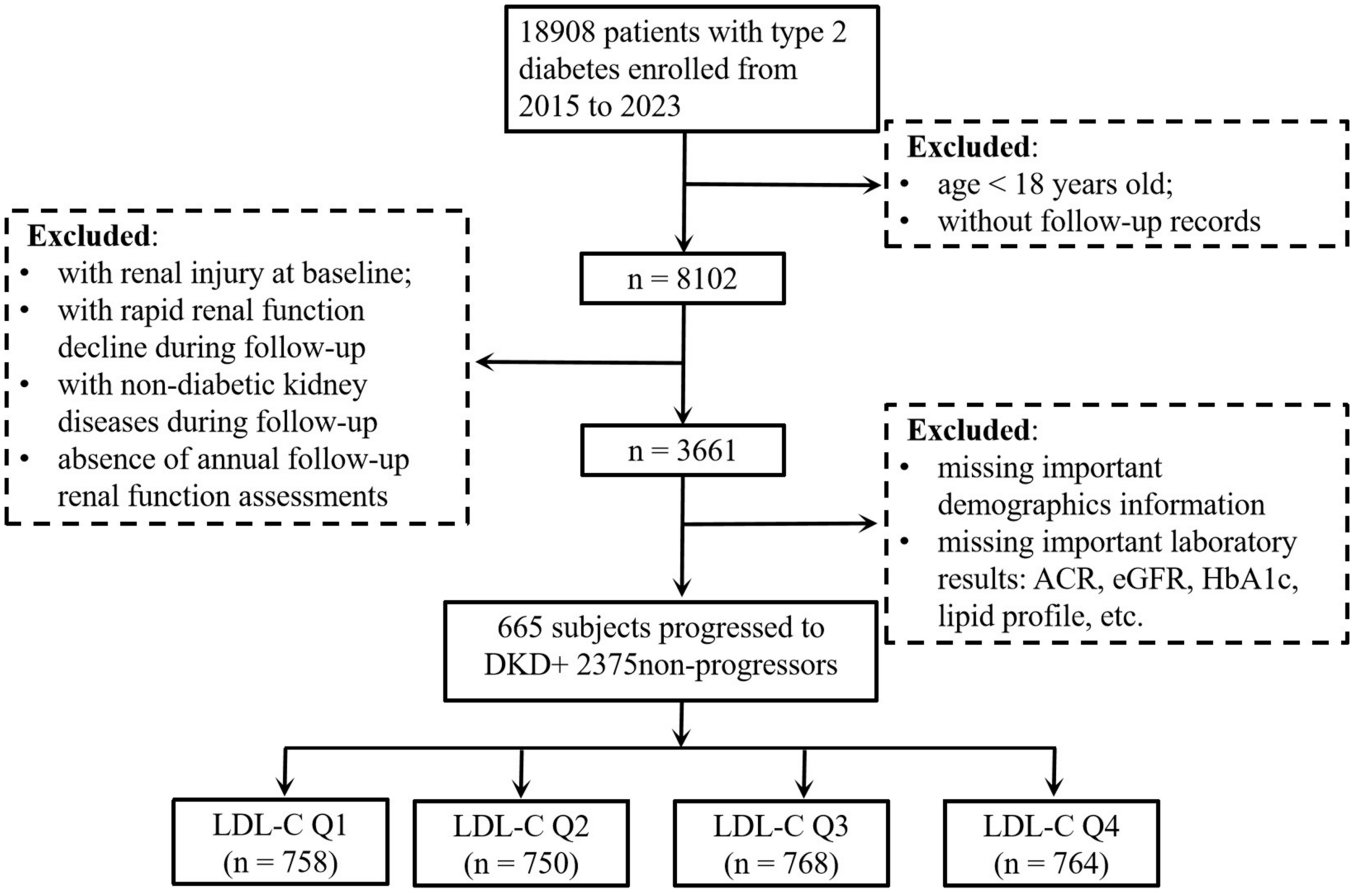
Flowchart of the subjects. eGFR, estimated glomerular filtration rate; ACR, albumin-to-creatinine ratio; DKD, diabetic kidney disease; HbA1c, glycated hemoglobin; LDL-C, low-density lipoprotein cholesterol.

This retrospective cohort study initially enrolled patients with T2D from the electronic health records of Affiliated Jinhua Hospital, Zhejiang University School of Medicine (2015–2023). During the selection process, patients were excluded based on the following criteria: (1) age <18 years, (2) baseline renal impairment defined as albumin-to-creatinine ratio (ACR) ≥ 30 mg/g or eGFR < 60 mL/min/1.73 m^2^, (3) lack of annual follow-up renal function assessments, (4) diagnosed as an acute kidney injury during follow-up according to the Kidney Disease: Improving Global Outcomes (KDIGO) criteria (≥50% serum creatinine increase within 7 days or absolute increase ≥0.3 mg/dL) (18), (5) diagnosed as non-diabetic kidney diseases (e.g., glomerulonephritis and polycystic kidney disease) during follow-up, and (6) incomplete critical clinical data (e.g., missing lipid profiles and renal injury markers). After applying these exclusions, the final cohort comprised 3,040 subjects with T2D and no baseline evidence of DKD. The participant flowchart is provided in Figure 1. This study followed the guidelines in the Declaration of Helsinki and was approved by the Ethics Committee of Affiliated Jinhua Hospital, Zhejiang University School of Medicine (ethical approval number: (Res) 2025-Ethical Review-163). According to the regulations of the Ethics Committee, informed consent was waived due to the retrospective nature of the study.

### 2.2 Data collection

Clinical data were extracted from the electronic health records of Affiliated Jinhua Hospital, Zhejiang University School of Medicine. Demographic and clinical parameters included age, sex, systolic blood pressure (SBP), diastolic blood pressure (DBP), body mass index (BMI), diabetes duration, and hypertension status. Medication usage at baseline was documented for angiotensin-converting enzyme inhibitors (ACEIs), angiotensin receptor blockers (ARBs), insulin, sodium-glucose cotransporter-2 inhibitors (SGLT2is), glucagon-like peptide-1 receptor agonists (GLP-1RAs), and lipid-lowering agents (statins and fibrates). Laboratory measurements encompassed lipid profiles [LDL-C, total cholesterol (TC), triglycerides (TG), and high-density lipoprotein cholesterol (HDL-C)], fasting blood glucose (FBG), glycated hemoglobin (HbA1c), serum creatinine (SCr), uric acid (UA), ACR, and eGFR. The value of eGFR was calculated using the Xiangya equation (19) for Chinese populations. In the present study, DKD was defined as ACR ≥ 30 mg/g and/or eGFR <60 mL/min/1.73 m^2^ during follow-up. Glycemic control was dichotomized as HbA1c ≤ 7.0% (good control) or >7.0% (poor control), based on the American Diabetes Association targets (20).

### 2.3 Statistical analysis

Continuous variables are presented as mean ± standard deviation or median (interquartile range), and categorical variables are presented as frequencies (percentages). Differences across LDL-C quartiles (Q1–Q4) were assessed using ANOVA, the Kruskal–Wallis test, or the chi-square test, as appropriate. The association between baseline LDL-C levels and DKD risk was evaluated using Cox proportional hazards models, with LDL-C analyzed both as a continuous variable and categorical quartiles. Multivariable adjustments were implemented hierarchically across three models: Model 1 was adjusted for age and sex; Model 2 extended adjustments to include SBP, BMI, HbA1c, diabetes duration, and hypertension status; Model 3 further incorporated baseline ACR, HDL-C, TG, eGFR, and use of SGLT2i/GLP-1RAs. Covariates were selected based on either (1) factors identified as DKD risk determinants in the previous studies (6, 21) or (2) variables with significant between-group differences (*p* < 0.05) in univariate analyses of this cohort.

Non-linear relationships were explored using restricted cubic splines (RCS). Interaction terms were tested to assess effect modification by glycemic control (HbA1c ≤ 7% vs. >7%), hypertension, sex, diabetes duration, and age. Stratified Cox models were applied to significant interaction subgroups. Sensitivity analyses excluded statin/fibrate users to assess robustness. In the well-controlled glycemic subgroup, inflection points for LDL-C thresholds were identified as the intersections of the RCS-derived hazard ratio (HR) curve with the null reference line (HR = 1). These thresholds were validated through piecewise regression and Kaplan–Meier survival curves with log-rank tests. All analyses used two-tailed tests (*p* < 0.05) and were conducted in R version 4.3.6 and SPSS 26.0.

## 3 Results

### 3.1 Clinical baseline characteristics of study participants

This study enrolled 3,040 T2D patients without baseline DKD. The mean age was 57.3 ± 11.58 years; 61.7% were male. The prevalence of hypertension was 45.4%, the median diabetes duration was 6 years, and the median follow-up time was 3.13 years. Patients were stratified into four groups (Q1–Q4) based on baseline LDL-C quartiles, with significant differences in baseline characteristics among all groups (Table 1). The lowest LDL-C group (Q1) exhibited significantly higher DKD progression rates than the other groups (*p* = 0.013), with the lowest risk observed in Q2. Patients in the highest LDL-C group (Q4) were younger (*p* < 0.001), had a lower proportion of male patients (57.9% vs. Q1: 66.0%; *p* = 0.013), and exhibited a decreasing trend in hypertension prevalence as LDL-C levels increased (*p* < 0.001). Additionally, HbA1c, FBG, TC, and TG levels all increased with higher LDL-C (all *p* < 0.001). For renal function, the highest LDL-C group exhibited higher baseline eGFR (*p* < 0.001), but no intergroup differences in ACR were observed (*p* = 0.299). Additionally, the lowest LDL-C group (Q1) showed higher usage rates of ACEI/ARB medications (*p* < 0.001) and statins/fibrates (*p* < 0.001), whereas SGLT2i/GLP-1RA medications were more frequently used in the highest LDL-C group.

**Table 1:** Baseline characteristics of the study population stratified by LDL-C quartiles.

Variables	LDL-C Q1	LDL-C Q2	LDL-C Q3	LDL-C Q4	*p*-value
	*n* = 758	*n* = 750	*n* = 768	*n* = 764	
Progressed to DKD, *n* (%)	196 (25.8)	143 (19.0)	163 (21.2)	163 (21.3)	0.013
Age, year	62.94 ± 10.69	58.25 ± 11.17	56.1 ± 11.19	54.92 ± 11.5	<0.001
Male, *n* (%)	501 (66.0)	464 (61.8)	469 (61.0)	443 (57.9)	0.013
Hypertension, *n* (%)	501 (66.0)	366 (48.8)	326 (42.4)	312 (40.8)	<0.001
SBP, mmHg	132.15 ± 18.25	134.19 ± 17.76	134.83 ± 18.41	133.23 ± 18.42	0.024
DBP, mmHg	75.82 ± 10.88	78.7 ± 10.88	79.81 ± 11.77	79.73 ± 11.13	<0.001
BMI, kg/m^2^	24.49 ± 4.28	24.56 ± 3.81	24.65 ± 4.43	24.72 ± 4.48	0.735
HbA1c, %	7.85 ± 2.07	8.22 ± 2.13	8.49 ± 2.23	8.85 ± 2.43	<0.001
TC, mmol/l	3.11 ± 0.74	4.01 ± 0.53	4.65 ± 0.43	5.72 ± 0.88	<0.001
LDL-C, mmol/l	1.82 ± 0.31	2.54 ± 0.16	3.07 ± 0.15	3.92 ± 0.56	<0.001
HDL-C, mmol/l	1.06 ± 0.29	1.11 ± 0.33	1.13 ± 0.29	1.21 ± 0.29	<0.001
TG, mmol/l	1.08 (0.84, 1.51)	1.37 (0.99, 1.97)	1.61 (1.15, 2.46)	1.82 (1.34, 2.54)	<0.001
FBG, mmol/l	6.92 ± 2.46	7.49 ± 2.68	7.88 ± 2.84	8.43 ± 3.11	<0.001
UA, μmol/L	314.27 ± 90.16	309.31 ± 84.77	314.9 ± 87.96	315.48 ± 89.82	0.507
Serum creatinine, μmol/L	75.95 ± 15.35	74.00 ± 13.94	74.10 ± 13.75	73.52 ± 13.14	0.004
eGFR, ml/min/1.73 m^2^	76.26 ± 9.03	78.27 ± 9.11	78.81 ± 8.78	79.22 ± 9.01	<0.001
ACR, mg/g	10.10 (4.59, 16.52)	9.54 (4.55, 16.30)	10.04 (5.40, 16.36)	10.24 (5.53, 16.58)	0.299
Diabetic duration, year	8 (3, 12)	6 (2, 10)	6 (2, 10)	5 (2, 10)	<0.001
Follow-up time, year	2.8 (1.47, 4.55)	3.22 (1.72, 5.05)	3.22 (1.80, 4.97)	3.30 (1.90, 5.30)	<0.001
History of drinking, *n* (%)	287 (37.8)	268 (35.7)	258 (33.5)	241 (31.5)	0.058
History of smoking, *n* (%)	295 (38.9)	290 (38.6)	265 (34.5)	264 (34.1)	0.114
Insulin therapy, *n* (%)	251 (33.1)	234 (31.2)	281 (36.5)	259 (33.9)	0.165
ACEI/ARB use, *n* (%)	204 (26.9)	128 (17.0)	132 (17.1)	115 (15.0)	<0.001
Fibrate/statin use, *n* (%)	228 (30.0)	91 (11.1)	80 (10.4)	109 (14.2)	<0.001
SGLT2i/GLP-1RA use, *n* (%)	89 (11.7)	59 (7.8)	88 (11.4)	102 (13.3)	0.007

DKD, diabetic kidney disease; SBP, systolic blood pressure; DBP, diastolic blood pressure; BMI, body mass index; HbA1c, glycated hemoglobin; TC, total cholesterol; LDL-C, low-density lipoprotein cholesterol; HDL-C, high-density lipoprotein cholesterol; TG, triglycerides; FBG, fasting blood glucose; UA, uric acid; eGFR, estimated glomerular filtration rate; ACR, albumin-to-creatinine ratio; ACEIs, angiotensin-converting enzyme inhibitors; ARBs, angiotensin receptor blockers; SGLT2is, sodium-glucose cotransporter-2 inhibitors; GLP-1RAs, glucagon-like peptide-1 receptor agonists.

### 3.2 Association between LDL-C levels and DKD risk in the overall population

Cox proportional hazards models evaluated the association between baseline LDL-C (as a continuous variable and quartiles) and DKD risk (Table 2). When LDL-C was analyzed as a continuous variable, no statistically significant association with DKD risk was observed in the fully adjusted model (per 1 mmol/L increase: HR = 0.91, 95% CI: 0.82–1.01, *p* = 0.061). When analyzed by quartiles, compared to the lowest quartile (Q1), Q2 showed a significantly reduced DKD risk (HR = 0.69, 95% CI: 0.55–0.87, *p* = 0.001), Q3 exhibited a partial rebound in risk (HR = 0.80, 95% CI: 0.64–1.00, *p* = 0.046), and Q4 demonstrated a subsequent decline (HR = 0.72, 95% CI: 0.58–0.91, *p* = 0.005). Although the trend test suggested a decreasing DKD risk with higher quartiles (*p* = 0.006), the non-monotonic HR pattern (lowest in Q2 → rebound in Q3 → decline in Q4) implies potential non-linearity. The RCS analysis further revealed that, despite no strong statistical evidence from the non-linearity test (P_non-linear_ = 0.075), DKD risk in the low LDL-C range decreased rapidly with increasing LDL-C, reaching the lowest risk, followed by only a mild upward trend with further LDL-C increases, resulting in a flattened curve (Supplementary Figure S1).

**Table 2:** Association between LDL-C and DKD risk via the Cox regression in the overall participants.

	Model 1			Model 2			Model 3		
LDL-C level	HR	95%CI	*P-*value	HR	95%CI	*P-*value	HR	95%CI	*P-*value
Per unit increase	0.93	0.84–1.02	0.112	0.89	0.81–0.98	0.016	0.91	0.82–1.01	0.061
Quartile 1	Ref			Ref			Ref		
Quartile 2	0.72	0.58–0.89	0.003	0.70	0.57–0.88	0.002	0.69	0.55–0.87	0.001
Quartile 3	0.82	0.66–1.02	0.068	0.82	0.66–1.02	0.068	0.80	0.64–1.00	0.046
Quartile 4	0.77	0.62–0.96	0.019	0.72	0.58–0.90	0.003	0.72	0.58–0.91	0.005
*P* for trend	0.017			0.006			0.006		

DKD, diabetic kidney disease; LDL-C, low-density lipoprotein cholesterol; HRs, hazard ratios; Quartile 1 of LDL-C: ≤ 2.25 mmol/L; Quartile 2 of HDL-C: 2.26–2.82 mmol/L; Quartile 3 of HDL-C: 2.83–3.35 mmol/L; Quartile 4 of HDL-C: > 3.35 mmol/L.

### 3.3 Interaction between LDL-C and glycemic control and stratified analyses

**Figure 2:**
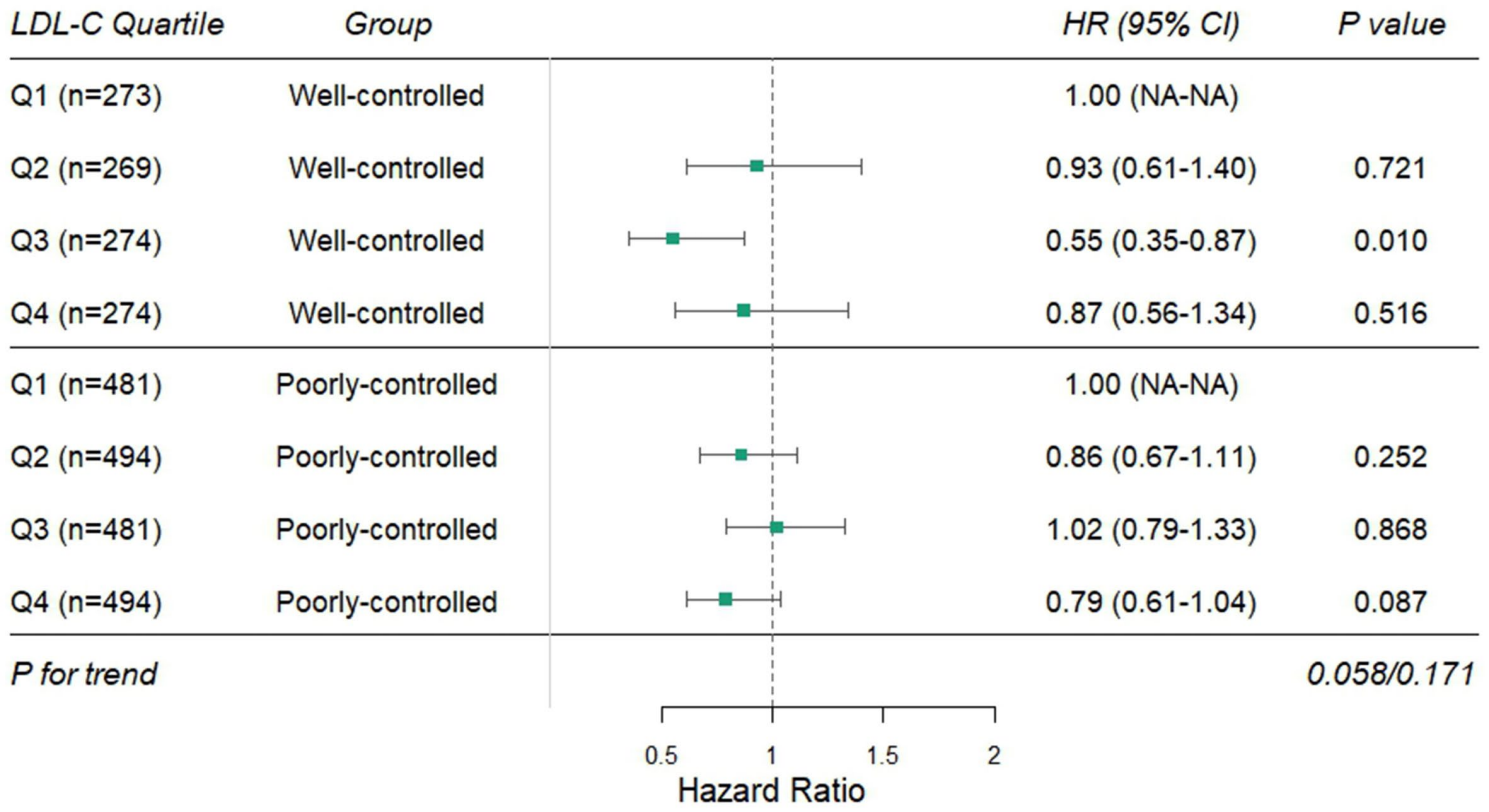
Stratified effects of LDL-C quartiles on DKD risk by glycemic control status. DKD, diabetic kidney disease; LDL-C, low-density lipoprotein cholesterol.

The Cox models were used to examine interactions between baseline LDL-C quartiles and glycemic control (HbA1c ≤ 7% vs. >7%), hypertension, sex, diabetes duration, and age, after adjusting for variables in Model 3 (Supplementary Table S1). The results showed that only glycemic control (HbA1c ≤ 7% vs. >7%) exhibited a significant interaction with LDL-C quartiles (interaction *p* = 0.013). In the well-controlled HbA1c subgroup (≤7%), the interaction term HR for Q3 was 0.47 (95% CI: 0.29–0.77, *p* = 0.003), suggesting heterogeneity in the LDL-C–DKD risk association depending on glycemic control status. However, interactions with sex, hypertension, diabetes duration, and age were not statistically significant (interaction *p* > 0.05). Stratified analyses by glycemic control were subsequently performed (Figure 2). In the well-controlled subgroup, compared to the lowest LDL-C quartile (Q1), Q3 showed a 45% reduction in DKD risk (HR = 0.55, 95% CI: 0.35–0.87, *p* = 0.010), while Q2 and Q4 exhibited no significant differences (all *p* > 0.05), indicating a potential non-linear association between LDL-C and DKD risk in this subgroup. In the poorly controlled subgroup, no significant differences in DKD risk were observed across LDL-C quartiles (all *p* > 0.05), indicating that the impact of LDL-C on DKD risk was significantly modulated by glycemic control.

### 3.4 RCS and sensitivity analysis in the well-controlled glycemic subgroup

**Figure 3:**
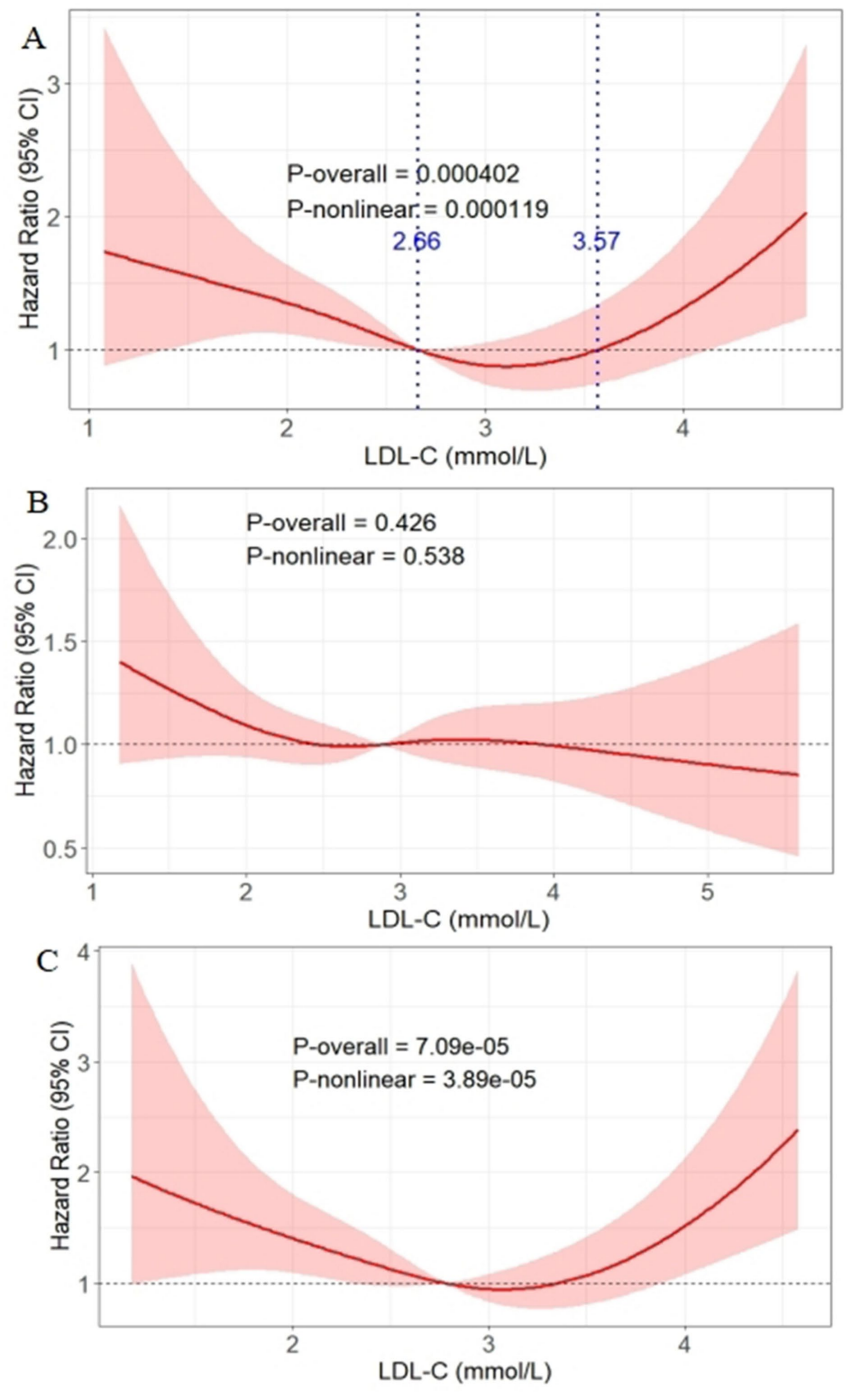
Association between LDL-C levels and DKD onset risk analyzed via RCS in the well-controlled glycemic subgroup **(A)**, poorly controlled subgroup **(B)**, and the well-controlled subgroup after excluding baseline statin/fibrate users **(C)**. The solid red line represents the hazard ratio for DKD; the shaded pink area represents the 95% confidence interval. The blue lines represent the curve intersecting the HR = 1 reference line at LDL-C = 2.66 mmol/L and 3.57 mmol/L. DKD, diabetic kidney disease; LDL-C, low-density lipoprotein cholesterol.

Based on the interaction results, RCS analysis was conducted in the well-controlled glycemic subgroup (HbA1c ≤ 7%, *n* = 1,090) to explore the dose–response relationship between LDL-C and DKD risk (Figure 3A). After adjusting Model 3, a significant U-shaped association was observed (P_overall_ < 0.001, P_non-linear_ < 0.001), with the curve intersecting the HR = 1 reference line at LDL-C = 2.66 mmol/L and 3.57 mmol/L. Specifically, when LDL-C was below 2.66 mmol/L, DKD risk decreased gradually with increasing LDL-C; the risk remained low within the 2.66–3.57 mmol/L range; and the risk increased with LDL-C levels above 3.57 mmol/L. Conversely, in the poorly controlled subgroup, no significant association was observed (P_overall_ = 0.426, P_non-linear_ = 0.538) (Figure 3B).

To exclude potential confounding by lipid-lowering medications, sensitivity analysis was performed in the well-controlled subgroup after excluding baseline statin/fibrate users (*n* = 865). The U-shaped association between LDL-C and DKD risk remained significant (P_overall_ < 0.001, P_non-linear_ < 0.001) (Figure 3C).

### 3.5 Validation of LDL-C threshold effects and survival analysis in the well-controlled glycemic subgroup

To validate the U-shaped threshold effects in the well-controlled subgroup, piecewise regression analysis was conducted (Supplementary Table S2). Using the LDL-C 2.66–3.57 mmol/L group as the reference, trend tests indicated increasing DKD risk with LDL-C levels deviating from the 2.66–3.57 mmol/L interval (trend test *p* = 0.043); the LDL-C < 2.66 mmol/L group showed a 55% increased DKD risk in the fully adjusted model (OR = 1.55, 95% CI: 1.08–2.20, *p* = 0.015), while the LDL-C > 3.57 mmol/L group exhibited a non-significant upward trend (OR = 1.47, 95% CI: 0.91–2.38, *p* = 0.121).

**Figure 4:**
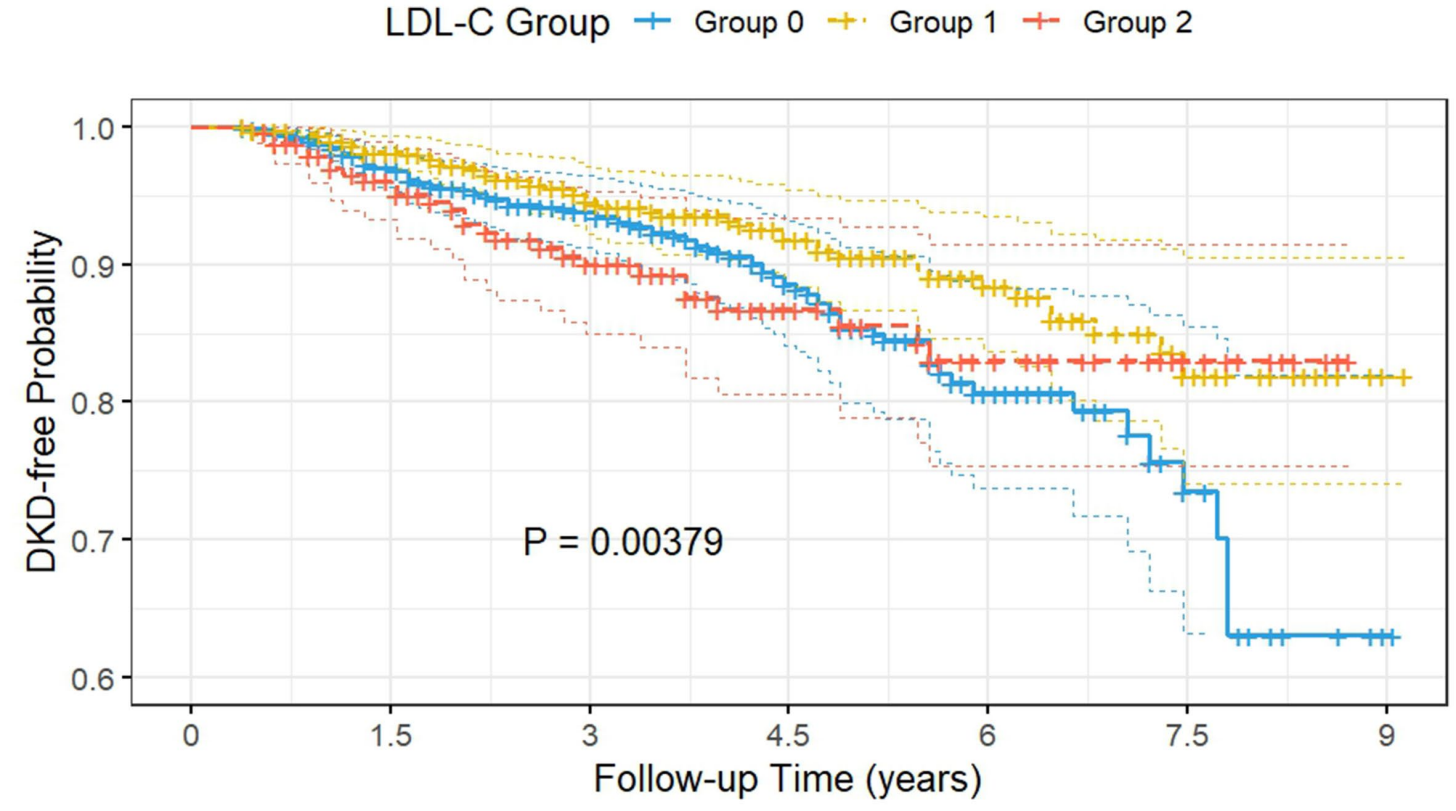
The Kaplan–Meier survival curves demonstrated that the intermediate LDL-C group had significantly lower cumulative DKD incidence compared to the low and high LDL-C groups. Group 0: subjects with low LDL-C (<2.66 mmol/L); Group 1: subjects with intermediate LDL-C (2.66–3.57 mmol/L); Group 2: subjects with high LDL-C (>3.57 mmol/L); DKD, diabetic kidney disease; LDL-C, low-density lipoprotein cholesterol.

Patients in the well-controlled subgroup were further categorized into three groups based on the thresholds: low LDL-C (<2.66 mmol/L), intermediate LDL-C (2.66–3.57 mmol/L), and high LDL-C (>3.57 mmol/L). The Kaplan–Meier survival curves (Figure 4) demonstrated that, after adjustment for Model 3, the intermediate LDL-C group had significantly lower cumulative DKD incidence compared to the low and high LDL-C groups (log-rank test *p* = 0.004).

## 4 Discussion

This retrospective cohort study of 3,040 patients with T2D without baseline DKD demonstrated a complex relationship between baseline LDL-C levels and DKD onset risk. In the overall cohort, LDL-C exhibited a non-monotonic relationship with DKD risk, with the lowest hazard observed in the second quartile. However, stratification by glycemic control uncovered a U-shaped relationship in patients with well-controlled glycemia (HbA1c ≤ 7%), where both low (<2.66 mmol/L) and high (>3.57 mmol/L) LDL-C levels were associated with elevated DKD risk, while intermediate levels (2.66–3.57 mmol/L) conferred the lowest risk. In contrast, no significant association was observed among individuals with poor glycemic control. These findings suggest that the relationship between LDL-C and DKD risk may vary by glycemic control status, emphasizing the need for tailored lipid management strategies in patients with T2D.

The pathogenesis of DKD involves intricate interactions between metabolic disturbances and renal injury (22). LDL-C, traditionally recognized for its atherogenic properties, exerts direct and indirect effects on renal physiology through multiple intersecting pathways (23). Experimental evidence suggests that native LDL particles infiltrate the glomerular basement membrane and renal tubulointerstitium, where local oxidative modification generates oxidized LDL (ox-LDL), a potent activator of innate immunity (24). Ox-LDL binds to scavenger receptors (e.g., LOX-1 and CD36) on podocytes and mesangial cells, triggering NF-κB-driven transcription of pro-inflammatory cytokines (e.g., IL-6 and TNF-α) and NLRP3 inflammasome assembly, thereby promoting macrophage recruitment and sustained renal inflammation (24–26). In the diabetic milieu, hyperglycemia amplifies these effects synergistically by enhancing LDL glycation, forming advanced glycation end product (AGE)-modified LDL, which exhibits stronger affinity for scavenger receptors and exacerbates oxidative stress (27, 28). Despite mechanism insights, clinical studies investigating the impact of LDL-C on DKD risk remain contentious (14–17). Our previous cross-sectional study identified a non-linear association between LDL-C levels and DKD prevalence (29). However, cross-sectional designs inherently limit causal inference, as they cannot establish temporal relationships between LDL-C exposure and DKD onset. To address this gap, we designed the current longitudinal cohort study to systematically explore the complex association patterns between LDL-C and DKD risk, leveraging time-to-event analyses to strengthen causal plausibility.

Our study found that, in the overall population, although the trend test for the association between LDL-C quartiles and DKD risk (*p* = 0.006) and RCS analysis (P_non-linear_ = 0.075) suggested no significant non-linear relationship between baseline LDL-C and DKD risk, the absence of a significant linear association when LDL-C was analyzed as a continuous variable (*p* = 0.061), the non-monotonic changes in hazard ratios across quartiles (lowest risk in Q2 → partial rebound in Q3 → decline in Q4), and the “descending-then-flattening” trend of the RCS curve collectively indicated potential non-linear association patterns not captured by traditional models. This complexity may stem from the bidirectional pathological effects of LDL-C on the kidney. Mechanistic studies demonstrate that excessively high LDL-C induces cholesterol overload in renal cells via CD36-mediated endocytosis, activating endoplasmic reticulum stress and the unfolded protein response, which triggers mitochondrial dysfunction and Bax/Bak-dependent apoptosis, ultimately promoting proteinuria and glomerulosclerosis (30–32). Conversely, extremely low LDL-C levels may disrupt the structural integrity of cholesterol-rich lipid rafts in renal cell membranes. These rafts serve as critical platforms not only for insulin receptor clustering and signaling (33, 34) but also for organizing podocyte survival signals and nephrin localization (35). Disruption of lipid rafts exacerbates insulin resistance in proximal tubules, impairing glucose reabsorption and creating a vicious cycle of metabolic dysregulation (33, 36). More importantly, in glomerular podocytes, raft disintegration may destabilize slit diaphragm complexes via diminished nephrin phosphorylation, directly promoting albuminuria and podocyte detachment (37). Additionally, LDL-C provides essential precursors for steroid hormone synthesis. Depletion of cholesterol substrate may reduce the production of vitamin D metabolites and anti-inflammatory glucocorticoids (30, 38). Vitamin D deficiency is linked to upregulated renal renin–angiotensin–aldosterone system activity and TGF-β1-driven fibrosis, while impaired glucocorticoid signaling attenuates the suppression of pro-inflammatory cytokines in renal tissues (39). Such dual impairment of steroidogenic pathways likely compromises the kidney’s adaptive response to oxidative and inflammatory insults, thereby accelerating DKD progression in the context of extreme hypocholesterolemia.

Based on the aforementioned hypotheses, this study revealed through interaction analysis that baseline LDL-C levels and glycemic control status (HbA1c ≤ 7% vs. >7%) had a significant interactive effect on DKD risk. Further subgroup stratification analysis demonstrated that, in T2D patients with well-controlled glycemia (HbA1c ≤ 7%), LDL-C exhibited a clear U-shaped association with DKD risk: the risk significantly increased when LDL-C was <2.66 mmol/L or >3.57 mmol/L, while maintaining LDL-C within 2.66–3.57 mmol/L conferred the lowest risk. While current guidelines advocate aggressive LDL-C reduction (<1.8 mmol/L) for cardiovascular protection (10, 11), our data suggest that, for T2D patients with optimal glycemic control, maintaining LDL-C at 2.66–3.57 mmol/L may optimize nephroprotection. This divergence underscores the necessity of disease-specific LDL-C targets. However, no significant association was observed in the subgroup with poor glycemic control (HbA1c > 7%). This phenomenon might be related to differences in the pathophysiological effects of LDL-C in different glycemic states. Previous studies have suggested that a hyperglycemic environment enhanced the renal toxicity of LDL by promoting LDL glycosylation modification and oxidative stress: AGE-modified LDL could be more readily taken up by glomerular mesangial cells, activating the NF-κB pathway and inducing the expression of pro-fibrotic factors, thereby accelerating glomerulosclerosis (40). Under well-controlled glycemia, the degree of LDL glycosylation was reduced, and its pro-inflammatory properties might be partially suppressed (40, 41); then, the bidirectional effects of LDL-C on the kidneys (low levels disrupting lipid raft signaling and high levels inducing endoplasmic reticulum stress) might become the dominant mechanisms. Additionally, the integrity of insulin signaling pathways might regulate LDL-C metabolism: insulin resistance accompanying hyperglycemia might impair the LDL receptor-mediated cholesterol clearance capacity in the liver, leading to a decoupling between elevated circulating LDL-C levels and renal LDL deposition (42, 43), thereby masking its association with DKD risk. These mechanisms collectively supported the findings of this study, indicating that glycemic control status was a critical modulator of the relationship between LDL-C and DKD risk.

This study has several limitations. First, as a single-center retrospective cohort study, the findings might lack generalizability to populations with different ethnic backgrounds or healthcare settings, necessitating validation through multicenter prospective studies. Second, reliance on baseline LDL-C measurements precluded assessment of cumulative LDL-C exposure and temporal variability, which could better reflect long-term lipid metabolism dynamics. Third, data on glycemic variability and hypoglycemia episodes were not available in our cohort, which may confound the interpretation of HbA1c-based stratification. Fourth, DKD was defined by ACR/eGFR thresholds, which may misclassify non-diabetic kidney disease or overlook early tubular injury, potentially attenuating the observed associations. Fifth, unmeasured confounders such as dietary lipid intake, genetic polymorphisms (e.g., APOE), and residual effects of prior lipid-lowering therapy could not be completely accounted for, which may bias the observed U-shaped association. Future studies should incorporate longitudinal lipid profiling, standardized multi-omics renal assessments, and comprehensive lifestyle data. More importantly, prospective interventional trials are warranted to validate whether individualized LDL-C targets can be translated into clinical practice.

## 5 Conclusion

In conclusion, our study demonstrated a significant interaction between LDL-C levels and glycemic control in influencing DKD risk among patients with type 2 diabetes. Specifically, a U-shaped relationship between LDL-C and DKD progression was observed exclusively in individuals with optimal glycemic control, suggesting potential limitations of the traditional “lower is better” approach to LDL-C management in this population. These findings indicate that maintaining LDL-C within an intermediate range may reduce DKD risk in patients with well-controlled glycemia, while no such association was evident in those with suboptimal glycemic control. This highlights the importance of considering glycemic status when evaluating LDL-C targets.

## Data Availability

The raw data supporting the conclusions of this article will be made available by the authors, without undue reservation.
