# Supplementary material for: Glycemic control modifies LDL-C–DKD risk: a U-shaped association in well-controlled type 2 diabetes
Source: Front Nutr. 2025 Sep 17;12:1660820. doi: 10.3389/fnut.2025.1660820 (PMC12483909; doi:10.3389/fnut.2025.1660820)
Supplement: Supplementary file 1 [file Supplementary_file_1.docx]

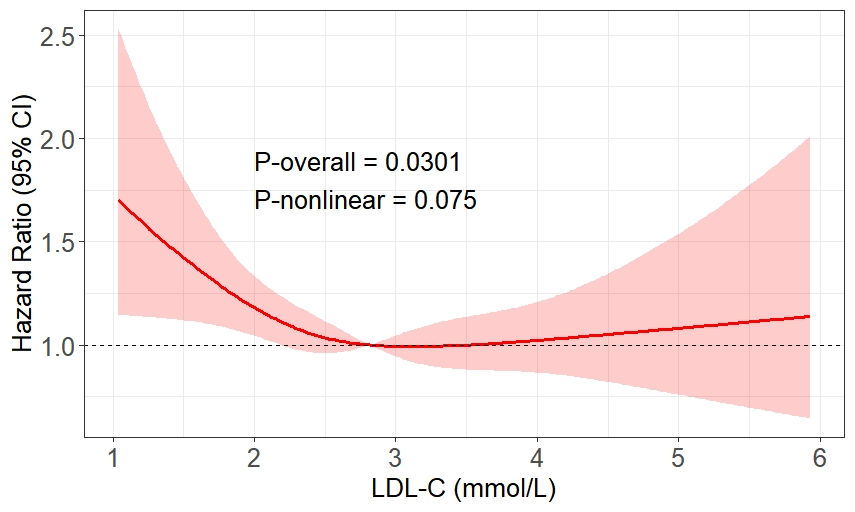


**Supplementary Figure S1. Association between LDL-C baseline levels and DKD risk analyzed performing by RCS in the overall study population.** DKD: diabetic kidney disease; RCS: restricted cubic spline; LDL-C: low-density lipoprotein cholesterol.

| Supplementary Table S1 Interaction effects of LDL-C quartiles with glycemic control, hypertension, and demographics on DKD risk | | | | |
| --- | --- | --- | --- | --- |
|  | HR | 95%CI | *P* value | *P*-interaction |
| Glycemic control (HbA1c > 7% vs ≤ 7%) |  |  |  |  |
| Glycemic control*LDL-C Q1 | Ref |  |  | 0.013 |
| Glycemic control*LDL-C Q2 | 0.99 | 0.62 - 1.59 | 0.964 |  |
| Glycemic control*LDL-C Q3 | 0.47 | 0.29 - 0.77 | 0.003 |  |
| Glycemic control*LDL-C Q4 | 0.94 | 0.59 - 1.48 | 0.776 |  |
| Gender (female vs male) |  |  |  |  |
| Gender*LDL-C Q1 | Ref |  |  | 0.650 |
| Gender*LDL-C Q2 | 1.07 | 0.68 - 1.67 | 0.779 |  |
| Gender*LDL-C Q3 | 0.81 | 0.53 - 1.24 | 0.331 |  |
| Gender*LDL-C Q4 | 0.98 | 0.64 - 1.50 | 0.927 |  |
| Hypertension (no vs yes) |  |  |  |  |
| Hypertension*LDL-C Q1 | Ref |  |  | 0.525 |
| Hypertension*LDL-C Q2 | 0.94 | 0.59 - 1.50 | 0.797 |  |
| Hypertension*LDL-C Q3 | 0.75 | 0.48 - 1.18 | 0.213 |  |
| Hypertension*LDL-C Q4 | 0.78 | 0.50 - 1.22 | 0.27 |  |
| Diabetic duration (< 10 vs ≥ 10 years) |  |  |  |  |
| Diabetic duration*LDL-C Q1 | Ref |  |  | 0.769 |
| Diabetic duration*LDL-C Q2 | 1.12 | 0.72 - 1.73 | 0.622 |  |
| Diabetic duration*LDL-C Q3 | 0.93 | 0.61 - 1.41 | 0.727 |  |
| Diabetic duration*LDL-C Q4 | 1.15 | 0.75 - 1.75 | 0.532 |  |
| Age (< 60 vs ≥ 60 years old) |  |  |  |  |
| Age grade*LDL-C Q1 | Ref |  |  | 0.637 |
| Age grade*LDL-C Q2 | 1.32 | 0.84 - 2.06 | 0.229 |  |
| Age grade*LDL-C Q3 | 1.22 | 0.80 - 1.88 | 0.357 |  |
| Age grade*LDL-C Q4 | 1.1 | 0.72 - 1.70 | 0.658 |  |

DKD: diabetic kidney disease; LDL-C: low-density lipoprotein cholesterol; HR: hazard ratios.

| Supplementary Table S2. Threshold effect analyses of LDL-C on the risk of DKD in the subjects with good glycemic control | | | | | | |
| --- | --- | --- | --- | --- | --- | --- |
| Variable | Model 1 | | Model 2 | | Model 3 | |
|  | OR (95% CI) | P value | OR (95% CI) | P value | OR (95% CI) | P value |
| LDL-C 2.66 - 3.57 mmol/L | 1.0 (refference ) | - | 1.0 (refference ) | - | 1.0 (refference ) | - |
| LDL-C: < 2.66 mmol/L | 1.47 (1.05 - 2.07) | 0.026 | 1.46 (1.03 - 2.05) | 0.032 | 1.55 (1.08 - 2.20) | 0.015 |
| LDL-C:﹥3.57 mmol/L | 1.39 (0.87 - 2.22) | 0.163 | 1.44 (0.90 - 2.29) | 0.130 | 1.47 (0.91 - 2.38) | 0.121 |
| P for trend | 0.078 | | 0.084 | | 0.043 | |

DKD: diabetic kidney disease; LDL-C: low-density lipoprotein cholesterol; HR: hazard ratios.
